# Organizational and Individual Interventions for Managing Work-Related Stress in Healthcare Professionals: A Systematic Review

**DOI:** 10.3390/medicina59101866

**Published:** 2023-10-20

**Authors:** Pierluigi Catapano, Salvatore Cipolla, Gaia Sampogna, Francesco Perris, Mario Luciano, Francesco Catapano, Andrea Fiorillo

**Affiliations:** Department of Psychiatry, University of Campania “Luigi Vanvitelli”, 80138 Naples, Italy

**Keywords:** stress, workplace, burnout, physician, healthcare workers, intervention

## Abstract

The workplace represents a relevant source of stress for workers, being a risk factor for many mental disorders and psychological difficulties, including burn-out syndrome. Healthcare workers and other help-professions are particularly susceptible to work-related stress. The present systematic review aims to (1) identify available interventions for managing workplace-related stress symptoms; (2) assess their efficacy; and (3) discuss the current limitations of available interventions. A systematic review has been conducted, searching on PubMed, APA PsycInfo, and Scopus databases. Eighteen papers have been identified, which included different interventions for the management of work-related stress in healthcare professionals. These approaches can be grouped as follows: (1) interventions focusing on the individual level using cognitive-behavioral therapy (CBT) approaches; (2) interventions focusing on the individual level using relaxation techniques; and (3) interventions focusing on the organizational level. As regards interventions targeting the individual level using CBT approaches, mindfulness-based interventions were effective in reducing levels of burn-out, stress, and anxiety and in improving quality of life. As regards intervention using relaxation techniques, including art therapy, Emotional Freedom Techniques (ECT) and brief resilience retreats had a positive effect on the levels of anxiety, stress, and burnout. As regards interventions at the organizational level, we found no evidence for supporting its effectiveness in reducing the levels of burnout. Furthermore, available studies are heterogeneous in terms of assessment tools, target populations, and type of interventions, which limits the generalizability of findings.

## 1. Introduction

Work-related stress is a complex phenomenon, which has been defined by the World Health Organization as “the response people may have when presented with work demands and pressures that are not matched to their knowledge and abilities and which challenge their ability to cope” [1]. Stress can affect workers in many different situations, and it is due to a lack of support from supervisors and other colleagues or to having little control over work processes [2]. The relationship between levels of stress and working performance is bidirectional: perceived pressure can be useful to keep the individual alert, motivated, able to work, and learn, but, when it exceeds a certain threshold—which varies among individuals—becomes excessive or unmanageable, causing stress. Stress can negatively influence employees’ health and their work performance. The workplace represents a relevant source of stress for workers due to excessive workloads, moral violence, work processes, interactions with patients’ families, professional and administrative demands, resource constraints, and lack of management support [3]. Therefore, workplaces can play a central role in the development of mental health problems—such as burn-out syndrome or full-blown mental disorders, mainly anxiety or depressive disorders. Burnout syndrome is composed of three dimensions of burnout: emotional exhaustion, depersonalization, and personal accomplishment [4]. The term “burnout” describes a physical and emotional strain specifically occurring in the work environment. Burnout syndrome is also known as chronic work-related stress syndrome [4,5,6,7,8,9,10].

As with other helping professions, healthcare workers are particularly susceptible to work-related stress due to the demands of their everyday clinical practice and the continuous exposure to patients’ suffering [11,12]. In particular, stress among healthcare workers ranges from 27 to 87.4% and it significantly affects their physical and mental health, risk of substance use, work-related delays, absenteeism, and presenteeism, as well as emigration rate [13]. Additionally, it can lead to patient safety concerns and poor quality of care. The mismatch between job requirements and available resources, work overload, working environment, work experience, workplace conflicts, gender discrimination, marital status, educational status, job satisfaction, and not being rewarded properly are some of the factors significantly associated with occupational stress among healthcare professionals. Moreover, an important element which can mediate the impact of job-related stress on the mental health of healthcare professionals is represented by the sense of coherence (SOC). This construct is defined as the general orientation of seeing life as understandable, manageable, and meaningful, and having the ability to cope with stressful situations. A recent systematic review by Pablo González-Siles (2022) has pointed out that stress, depression, burnout, and posttraumatic stress disorder (PTSD) negatively correlate with SOC; in contrast, job satisfaction, well-being, and quality of life positively correlate with SOC [14]. 

The coronavirus disease 2019 (COVID-19) pandemic introduced additional stressors, such as staff redeployment and fear of infection [15]. During the pandemic, increased levels of work-related stress and of burn-out symptoms have been reported by healthcare professionals [16], due to the need to manage an unexpected public health emergency without adequate knowledge and safety means [17,18,19,20,21,22,23]. According to EU-OSHA’s workers’ survey OSH Pulse—occupational safety and health in post-pandemic workplaces---almost half of healthcare workers (44%) reported that their work stress had increased in particular during the first wave of the pandemic [24,25,26,27,28,29].

In the initial phase of the pandemic, levels of stress, anxiety symptoms, and sleep difficulties increased in first-line professionals [30,31,32]. Also, suicidal risk and suicidal ideation initially increased [33], with a further worsening of the burden associated with workplace stress [34,35,36,37]. Therefore, the need to develop supportive interventions for promoting the mental health of healthcare workers has been advocated, and, actually, many hospitals dealing with COVID-19 patients have established helplines or promoted psychological help for all healthcare professionals in need of some support [38,39,40].

Work-related stress on mental health has been recognized as one of the most powerful stressful events for mental health by the World Health Organization and by several international scientific associations, such as the World Psychiatric Association and the European Psychiatric Association, who are committed to implement and disseminate strategies and multilevel interventions for the prevention, early detection, and management of work-related stress symptoms. 

Interventions for work-related stress symptoms can be grouped into those focusing on the individual level, using cognitive-behavioural techniques or relaxation approaches, and those focusing on the organizational level [41].

The present systematic review aims to (1) identify available individual or organization-level interventions for the management of workplace-related stress symptoms; (2) assess their efficacy; and (3) discuss the eventual limitations of the available interventions.

## 2. Methods

### 2.1. Search Strategy

An extensive literature search for relevant articles has been performed on PubMed, APA PsycInfo, and Scopus databases entering the following terms: “Occupational Stress” [Mesh] AND (“Anxiety” [Mesh] OR “Depression” [Mesh] OR “Stress, Physiological” [Mesh]) AND (“Health Personnel” [Mesh] OR “Health Care Facilities, Manpower, and Services” [Mesh]) AND (“prevention and control” [Subheading] OR prevention [Text Word]), using “Abstract”, “Humans”, “English” as filters on Pubmed; (TITLE-ABS-KEY (occupational AND stress AND (anxiety AND depression AND stress)) AND ((health AND personnel) OR (health AND care AND facilities AND manpower AND services)) AND (prevention) AND PUBYEAR > 2014 AND PUBYEAR < 2024AND (LIMIT-TO (DOCTYPE, “ar”)) AND (LIMIT-TO (LANGUAGE, “English”))), on Scopus; (abstract(occupational stress) AND noft (anxiety) AND noft (depression) AND noft (stress) AND abstract (health personnel) OR abstract(health care facility) OR abstract (health care facilities) AND noft (prevention)) on APA PsycInfo.

The search method has been conducted according to the Preferred Reporting Items for Systematic Review and Meta-Analysis (PRISMA) statement, as applicable [42]. 

### 2.2. Selection Criteria

The literature search was limited to the period from 2015 up to May 2023, since studies previously published are already covered in the review by Ruotsalainen et al. [41]. Only papers written in English were included. The reference lists of included articles were screened to identify additional relevant studies. The following inclusion criteria were used: (1) studies involving medical doctors, nurses, health personnel or medical students, student nurses or physicians in training; (2) studies describing interventions aiming to prevent or reduce work-related stress; and (3) studies reporting occupational and work-related stress or burnout levels as outcomes. Only studies reporting work-related stress evaluated at the individual/personal level were included.

### 2.3. Selection Process

**Figure 1:**
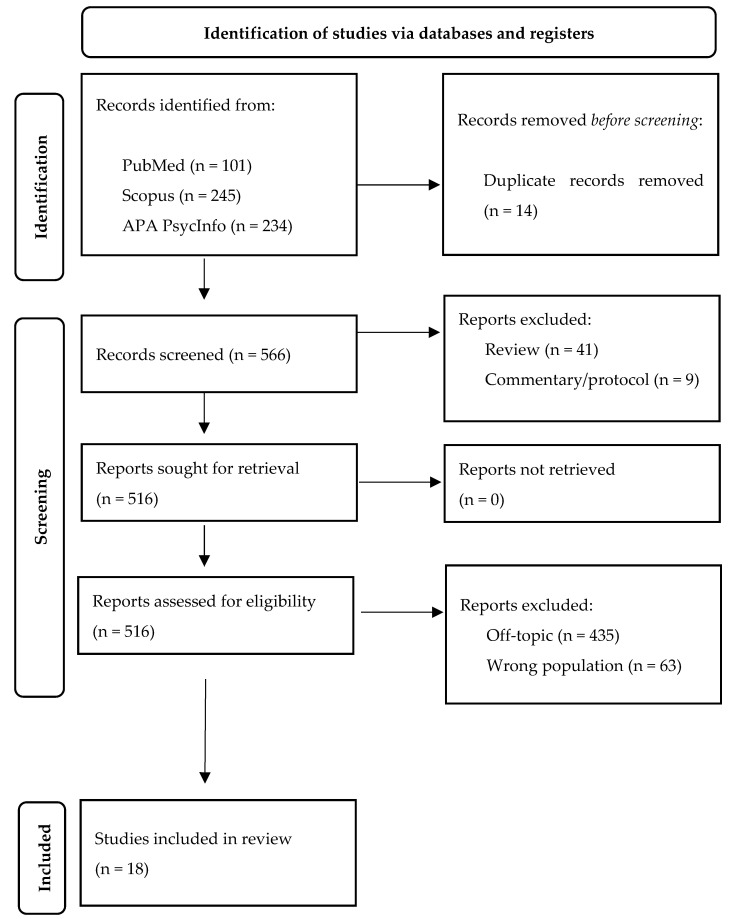
Flowchart of the included studies.

A total of 580 papers were identified, 14 papers were duplicates and were removed, while the remaining (N = 548) were excluded because they were not relevant. Therefore, N = 18 papers were evaluated in detail and included in the analysis (Figure 1).

SC and PC extracted the relevant data and synthetized them in a tabular format; GS and FP triple-checked the extracted data for accuracy. 

Inter-rater reliability, referring to the degree of agreement between researchers, has been calculated, with a Cohen’s kappa score of 0.9.

### 2.4. Risk-of-Bias Assessment

Two authors (SC and PC) independently evaluated each selected study for the risk of bias, using the criteria recommended for Randomized Clinical Trials (RCTs) in the Cochrane Handbook for Systematic Reviews of Interventions [43] and the recommended tool for assessing risk of bias in non-randomized studies of intervention (NRSI) called ROBINS-I [44], p. 25.

The overall risk of bias was rated as moderate to high in all non-randomized studies included in the review; Supplementary Table S1 shows the considered domains and sub-domains. Two authors resolved disagreements through discussion or by involving a third author (GS). Results of risk of bias assessment for the RCTs are reported in Supplementary Table S2. One study was considered to be at a low risk of bias.

## 3. Results

Based on Ruotsalainen et al.’s seminal work [41], included studies have been grouped in three categories: (1) studies focusing on the individual level using cognitive-behavioural therapy approaches (Table 1); (2) studies focusing on the individual level using relaxation techniques (Table 2); and (3) studies focusing on the organizational level (Table 3).

The most frequently used tools to assess burnout, work-related stress, its impact on quality of life, and psychopathological and psychological symptoms were the Maslach Burnout Inventory (MBI) for burnout (6 out of 18 studies) [45,46,47,48,49,50]; the Professional Quality of Life Scale (Pro-QOL) [51,52] and the Satisfaction With Life Scale (SWS) [46,49] for quality of life; the Perceived Stress Scale (PSS) [46,47,48,53,54]; the State and Trait Anxiety Inventory (STAI) [46,48,49,55,56]; the Cognitive and Affective Mindfulness Scale—Revised (CAMS-R) [47,48]; and the Patient-Reported Outcomes Measurement Information System (PROMIS) [50,53] for the assessment of stress and clinical symptoms. The tools used in each study, grouped by category, are reported in Table 4.

### 3.1. Studies Focusing on the Individual Level Using Cognitive-Behavioural Therapy or Other Psychotherapeutic Approaches

A total of 11 studies (out of 18) were focused on reducing the individual levels of stress and burn-out using CBT or other psychotherapeutic approaches (Table 1). Studies were carried out mainly in the USA and Spain, with sample sizes ranging from 13 participants in the study by dos Santos et al. [46] to 105 healthcare workers in Montaner et al. [49]. Included participants were mainly nurses (N = 8 studies) [44,45,48,51,53,56,57,58], other healthcare workers (N = 4 studies) [49,57,59,60], physicians (N = 3 studies) [49,54,57], and trainees in healthcare professions (N = 2 studies) [48,51]. Interventions provided to participants included a cognitive-behavioural component, ranging from mindfulness-based programmes [45,46,54,59,60] to informative interventions [48,51].

Many different assessment tools have been adopted for evaluating the levels of burn-out and stress reported by professionals, including the Maslach Burnout Inventory (MBI) [45,46,48,49], Perceived Stress Scale (PSS) [46,48,54], Depression, anxiety, and stress scale (DASS-21) [51,60], Copenhagen Burnout Inventory (CBI) [60], Distress Thermometer Assessment (DTA) [45], The World Health Organization Health and Work Performance Questionnaire (HPQ) [45], and Work Stress Scale (WSS) [46]. Tools for assessing quality of life included the Professional Quality of Life Scale (ProQOL) [51,52], Satisfaction With Life Scale (SWLS) [46,49], EuroQol (EQ-5D) [45], General Health Questionnaire (GHQ) [57], and WHO Quality of Life-BREF (WHOQOL-BREF) [46] (Table 4). Studies [46,54,59,60] confirm that mindfulness-based interventions are effective in the short-term in reducing levels of burn-out, stress, and anxiety and in improving quality of life. However, longitudinal studies—such as those by dos Santos et al. and by Haghighinejad et al. [46,60]—showed that the effect was not sustained at two/three months of follow-up. Furthermore, in two RCTs [45,51] group stress management programs were not effective in reducing the levels of burnout and work-related stress compared to the control group. No significant differences were found in other secondary outcomes, such as risk of depression and alcohol abuse. 

In the study by Tarquinio et al. [58], carried out during the COVID-19 pandemic, at-a-distance EMDR intervention provided to healthcare professionals previously treated with the same in-person technique was effective in reducing the levels of stress, although data on the long-term effectiveness are not available. Encouraging results were obtained from clinical trials using humor-based training sessions, stress-resilience courses, and Acceptance and Commitment Therapy (ACT) [48,49,57]. On the other hand, the reduction in secondary traumatic stress obtained by Sullivan throughout self-care practices was not confirmed at the 6-month follow-up [52].

### 3.2. Studies Focusing on the Reduction in the Levels of Stress and Burn-Out Using Relaxation Techniques

Five studies, mainly from the US, were included, with sample sizes ranging from 34 to 106 participants [53,61], who were mainly nurses (N = 4 studies) [53,55,56,61], trainees in healthcare professions (N = 3 studies) [53,61,62], and other healthcare workers (N = 3 studies) [53,61,62] (Table 2). Only the study by Kaimal et al. [53] included family caregivers. The interventions provided to participants shared relaxation techniques, such as breathing sessions [55], art therapy [53], acupressure points stimulation [56], group retreats [61], and nature and forest therapy [62].

Studies using art therapy, Emotional Freedom Tecniques (ECT), and brief resilience retreats [53,56,61] had a positive effect on the levels of anxiety, stress, and burnout (Table 4). However, these positive effects were not confirmed by biological markers, such as the salivary levels of IL-6, cortisol, and CRP [53]. Other relaxation practices, such as Shinrin-Yoku [62] and Relaxation Response [55], did not yield any significant effect.

### 3.3. Studies Focused on Organizational Level

Two studies, both carried out in the US, were included, with a total of 221 participants, mainly physicians [50], residents, and clinical fellows [47], not all engaged in clinical activities (Table 3). Lebares et al., who used a mixed-intervention program including the application of mindfulness in association with organizational initiatives, found that the integrated intervention reduced negative emotions and improved workplace satisfaction [47]. However, the only RCT involving an organizational intervention found no evidence for supporting its effectiveness in reducing levels of burnout [50].

**Table 1:** Studies focused on individual level using CBT or other psychotherapeutic approaches.

First Author (Publication Year)	Type of Study (Country)	Treatment Arms	Intervention	Outcome Measures of Effectiveness	Results
dos Santos T.M. (2015) [46]	Clinical Trial (Brazil)	13 nurses	Stress Reduction Program (SRP), based on the Mindfulness-Based Stress Reduction program	PSS; MBI; BDI; STAI; SWLS; SCS; WHOQOL-BREF; WSS	Results suggest that a SRP involving mindfulness may be a potentially effective approach to improve stress, depression, and QoL in a hospital setting. Comparison between pre- and post-intervention revealed a significant reduction in scores for perceived stress-PSS (*p* = 0.001), burnout-MBI (*p* = 0.02), depression-BDI (*p* = 0.007), and anxiety trait-STAI (*p* = 0.049), and a significant increase in scores for physical (*p* = 0.002) and psychological (*p* = 0.007) domains of the quality-of-life scale. These values remained stable six weeks after the intervention, except for the physical and psychological domains of the QoL scale, which showed a significant decline at follow-up (*p* < 0.05).
Axisa C. (2019) [51]	Randomized Controlled Trial (Australia)	46 physician trainees (23 intervention group; 23 control group)	Workshop to outline strategies for wellbeing and stress management	AUDIT; DASS-21; ProQOL	There was a small but not statistically significant reduction in alcohol use (*p* = 0.23), depression (*p* = 0.49), and burnout (*p* = 0.83) in the intervention group compared to the control group, measured at the primary endpoint at 6 months.
Luzarraga J. (2019) [59]	Clinical Trial (USA)	Pediatric Respiratory Therapists (40 first session, 24 second session)	Mindfulness-based intervention as part of staff meeting	DTA; breath rate	Integration of mindfulness-based interventions as part of staff meetings decreased members’ physical and emotional stress-related symptoms and increased members’ sense of calm. The distress scores were noted to decrease in session 1 (*p* = 0.001) and in session 2 (*p* = 0.39). Breathing rate also decreased during both sessions (*p* = 0.001).
Rinaldi A. (2019) [54]	Clinical Trial (Italy)	7 physicians and 13 nurses	Mindfulness intervention called Focus	PSS-10	There was a significant reduction in perceived stress (*p* = 0.019) from baseline to the end of the course.
Sullivan C.E. (2019) [52]	Clinical Trial (USA)	37 nurses in a pediatric oncology unit	Organizational and individual self-care practices	ProQOL; Brief COPE; CD-RISC2	A statistically significant reduction in secondary traumatic stress (*p* = 0.029) was found comparing pre-intervention and 4-month post-intervention scores; these data were not confirmed after the 6-month follow-up period, probably due to concomitant holidays (recognized as stress factor).
Watanabe N. (2019) [45]	Randomized Controlled Trial (Japan)	80 junior nurses (40 intervention group; 40 control group)	Group brief mindfulness-based stress management program (the control group received a psychological brochure)	HADS; PRIME-MD; GAD-7; MBI; ISI; HPQ; EQ-5D	No significant differences between the program and leaflet groups were observed in all the outcomes.
León-Pérez J.M. (2021) [57]	Clinical Trial (Spain)	58 medical and non-medical staff in an emergency ambulance service	13 humor-based training session (like social skills training procedures)	MSHS; STCI-S; GHQ-28	After-training scores were higher in positive attitudes toward humor (*p* = 0.001) and cheerfulness (*p* = 0.001) and lower in seriousness (*p* = 0.001) and psychological distress in almost all its dimensions (*p* < 0.05). After training, it was observed a reduction of 10-5% of potential cases of minor psychiatric disorder (from 62.1% to 51.7% using a 6-point cut-off in GHQ; from 55.2% to 50% using a 7-point cut-off).
Luton O.W. (2021) [48]	Clinical Trial (UK)	38 core surgical trainees (14 completed the course; 10 discontinued the intervention; 14 declined to participate)	5 weeks enhanced stress-resilience training (ESRT) course	MBI; PSS-10; PHQ-2, CAMS-R; STAI	Data analysis compared results between intervention group and those who declined to participate in the course. No significant differences were found between groups except for the levels of perceived stress: the median score at PSS-10 was lower (range 8–33) in the intervention group than in the non-intervention group (11–34) (*p* < 0.01).
Montaner X. (2021) [49]	Randomized Controlled Trial (Spain)	105 healthcare workers with patients affected by cognitive impairment and/or dementia (51 intervention group; 54 control group)	Acceptance and Commitment Therapy (ACT)	AAQ-II; MBI; STAI; SWLS	ACT intervention was effective in reducing anxiety (*p* < 0.001) and emotional exhaustion (*p* < 0.01) and in increasing the life satisfaction (*p* < 0.001) and personal accomplishment (*p* < 0.001) of workers, maintained at the 3 and 12-month follow-up. There were no differences in psychological flexibility between the intervention and control group.
Tarquinio C. (2021) [58]	Clinical Trial (France)	17 nurses facing patients with COVID-19, who was already in EMDR therapy	Remote Eye Movement Desensitization and Reprocessing (EMDR) therapy treatment	HADS; SUD	There was a significant (*p* < 0.001) decrease in the anxiety score, the depression score, as well as in the SUD. The scores then appear to be stable over time between the post-test after 24 h and 1 week later.
Haghighinejad H. (2022) [60]	Randomized Controlled Trial (Iran)	50 non-medical hospital staff (25 intervention group; 25 control group)	Modified mindfulness-based stress reduction (MBSR) program	CBI; DASS-21	Immediately after the training, the results showed that the reduction in burnout in dimensions of “work-characteristic-related”, “client-related” and “work-distaste-related” and decreased anxiety and stress scores in the intervention group were significantly more than in the control group (*p* < 0.05). After 3 months this effect was sustainable, although the downward trend in reducing the average burnout score had not continued.

AAQ-II: Acceptance and Action Questionnaire-II; AUDIT: Alcohol Use Disorders Identification Test; BDI: Beck Depression Inventory; Brief-COPE: Coping Orientation to Problems Experienced Inventory; CAMS-R: Cognitive and Affective Mindfulness Scale – Revised; CBI: Copenhagen Burnout Inventory; CD-RISC2: Connor-Davidson Resilience Scale-2; DASS-21: Depression, anxiety, and stress scale; DTA: Distress Thermometer Assessment; EQ-5D: EuroQol; GAD-7: Generalized Anxiety Disorder Scale; GHQ: General Health Questionnaire; HADS: Hospital Anxiety and Depression Scale; HPQ: The World Health Organization Health and Work Performance Questionnaire; ISI: Insomnia Severity Index; MBI: Maslach Burnout Inventory; MSHS: Multidimensional Sense of Humor Scale; PHQ-2: Patient Health Questionnaire; PRIME-MD: Primary Care Evaluation of Mental Disorder; ProQOL: Professional Quality of Life Scale; PSS: Perceived Stress Scale; QoL: Quality of Life; SCS: Self-Compassion Scale; STAI: State and Trait Anxiety Inventory; STCI-S: State-Trait Cheerfulness Inventory; SUD: Subjective Units of Distress Scale; SWLS: Satisfaction With Life Scale; WHOQOL-BREF: WHO Quality of Life-BREF; WSS: Work Stress Scale.

**Table 2:** Studies focused on individual level using relaxing techniques.

First Author (Publication Year)	Type of Study (Country)	Treatment Arms	Intervention	Outcome Measures of Effectiveness	Results
Calder Calisi C. (2017) [55]	Randomized Controlled Trial (USA)	46 nurses (24 intervention group; 22 control group)	Training on the technique of the Relaxation Response (RR) and practice the RR over an 8-week period	STAI; semantic differential scales	The mean change in the scores from baseline to post assessment did not differ significantly between groups.
Kaimal G. (2019) [53]	Clinical Trial (USA)	34 professional (n = 25) and informal (n = 9) caregivers	Two brief art-making interventions: open studio art therapy or the active control coloring session	PANAS; PSS; GSE; SSCS; PROMIS; MBI; cortisol, IL-6 and CRP levels in saliva samples	Caregivers in both interventions demonstrated improvements across all psychological outcomes (creative agency, *p* < 0.001; self-efficacy, *p* = 0.015; positive affect, *p* < 0.001; negative affect, *p* < 0.001; perceived stress, *p* < 0.001; anxiety, *p* = 0.002; burnout, *p* = 0.041). There was no evidence of change in any of the biomarkers (cortisol, IL-6, and CRP) measured.
Dincer B. (2020) [56]	Randomized Controlled Trial (Turkey)	72 nurses caring for COVID-19 patients (35 intervention group; 37 control group)	On-line brief single-session group intervention utilizing Emotional Freedom Techniques (EFT)	SUD; STAI; Burnout Scale	In the intervention group, there was a statistically significant reduction in stress levels (*p* < 0.001), anxiety levels (*p* < 0.001), and burnout levels (*p* < 0.010). No differences between pre- and post-intervention tests were found in the control group.
Cunningham T. (2021) [61]	Clinical Trial (USA)	106 healthcare professionals	Ten group sessions of daylong resilience retreats	19-item survey developed by the research team	There was a statistically significant decrease in state anxiety scores following the retreat (*p* < 0.001). Brief resilience retreats can reduce perceived anxiety and facilitate engagement in contemplative practices, which are associated with a decrease in the risk of burnout.
Kavanaugh J. (2022) [62]	Randomized Controlled Trial (USA)	34 health science faculty and medical resident (24 intervention group; 10 control group)	Guided forest bathing session (Shinrin-Yoku)	OLBI; Mini-Z	The data from this randomized controlled trial did not demonstrate a change in burnout symptoms from participating in a single Shinrin-Yoku walk when compared to baseline burnout scores or when compared to a control group.

GSE: General Self-Efficacy Scale; MBI: Maslach Burnout Inventory; Mini-Z: Work-related burnout symptoms questionnaire modified from Minimizing Error Maximizing Outcome; OLBI: Oldenburg Burnout Inventory; PANAS: Positive and Negative Affect Schedule; PROMIS: Patient-Reported Outcomes Measurement Information System; PSS-10: Perceived Stress Scale; SSCS: Short Scale of Creative Self; STAI: State and Trait Anxiety Inventory; SUD: Subjective Units of Distress Scale.

**Table 3:** Studies focused on organizational level.

First Author (Publication Year)	Type of Study	Treatment Arms	Intervention	Outcome Measures of Effectiveness	Results
West C.P. (2021) [50]	Randomized Controlled Trial (USA)	123 physicians (61 control group; 62 intervention group)	Self-facilitated physician small-group meetings	PJSS; EWS; QOL single question; MBI; PRIME-MD; SPS; PROMIS Social Isolation Short form 4a Scale	No statistically significant differences were seen in mean changes in burnout scale scores, meaning, or social support, although numeric differences generally favored the intervention.
Lebares C.C. (2021) [47]	Clinical Trial (USA)	64 trainees (residents and clinical fellows) in Department of Surgery	Individual and organizational-level initiatives, including mindfulness-based affective regulation training, advanced scheduling of time off, wellness half-days, and the creation of a resident-driven well-being committee	MBI, PSS, PHQ-2, AUDIT, STAI, CAMS-R, MHC-SF, DCSQ	Results reflected the potency of both social support and affective regulation skills in their ability to mitigate negative emotional influences on trainee work satisfaction.

AUDIT: Alcohol Use Disorders Identification Test; CAMS-R: Cognitive and Affective Mindfulness Scale – Revised; DCSQ: Demand-Control-Support Questionnaire; EWS: Empowerment at Work Scale; MBI: Maslach Burnout Inventory; MHC-SF: Mental Health Continuum–Short Form; PHQ-2: Patient Health Questionnaire; PJSS: Physician Job Satisfaction Scale PRIME-MD: Primary Care Evaluation of Mental Disorder; PROMIS: Patient-Reported Outcomes Measurement Information System; PSS-10: Perceived Stress Scale; QoL: Quality of Life; SPS: Social Provisions Scale; STAI: State and Trait Anxiety Inventory.

**Table 4:** Assessment tools used for each study. Copenhagen Burnout Inventory (CBI); The Swedish Demand -Control -Support Questionnaire (DCSQ); Early Warning Score—EWS; General Self-Efficacy scale (GSE); Health professional questionnaire (HPQ); Maslach Burnout Inventory (MBI); oldenburg burnout inventory (OLBI); Physician Job Satisfaction Scale (PJSS); Work Stressors Scale (WSS); General Health Questionnaire-28 (GHQ-28); Mental Health Continuum Short Form (MHC-SF); Professional Quality of Life (proQOL); Satisfaction With Life Scale (SWLS); Beck Depression Inventory (BDI); Depression Anxiety Stress Scales Short Version (DASS-21); General Anxiety Disorder-7 (GAD-7); Hamilton Depression Rating scale (HAD-S); Insomnia severity index (ISI); Patient Health Questionnaire (PHQ-2); Perceived Stress Scale (PSS); The State-Trait Anxiety Inventory (STAI).

	Group I											Group II					Group III	
	dos Santos (2015) [46]	Axisa (2019) [51]	Luzarraga (2019) [59]	Rinaldi (2019) [54]	Sullivan (2019) [52]	Watanabe (2019) [45]	León-Pérez (2021) [57]	Luton (2021) [48]	Montaner (2021) [49]	Tarquinio (2021) [58]	Haghighinejad (2022) [60]	Calder Calisi (2017) [55]	Kaimal (2019) [53]	Dincer (2020) [56]	Cunningham (2021) [61]	Kavanaugh (2022) [62]	West (2021) [50]	Lebares (2021) [47]
Burnout assessment																		
*19 item survey*															+			
*Burnout scale*														+				
*CBI*											+							
*DCSQ*																		+
*EWS*																	+	
*GSE*													+					
*HPQ*						+												
*MBI*	+					+		+	+								+	+
*Mini-Z*																+		
*OLBI*																+		
*PJSS*																	+	
*WSS*	+																	
Quality of life assessment																		
*EQ-5D*						+												
*GHQ-28*							+											
*MHC-SF*																		+
*Pro-QOL*		+			+													
*QOL single question*																	+	
*SWLS*	+								+									
*WHOQOL-BREF*	+																	
Psychiatric assessment																		
*AUDIT*		+																+
*BDI*	+																	
*DASS-21*		+									+							
*DTA*			+															
*GAD-7*						+												
*HADS*						+				+								
*ISI*						+												
*PHQ-2*								+										+
*PRIME-MD*						+											+	
*PSS*	+			+				+					+					+
*STAI*	+							+	+			+		+				
*SUD*										+				+				
Psychological assessment																		
*AAQ-II*									+									
*Brief-COPE*					+													
*CAMS-R*								+										+
*CD-RISC2*					+													
*MSHS*							+											
*PANAS*													+					
*PROMIS*													+				+	
*SCS*	+																	
*Semantic scale*												+						
*SPS*																	+	
*SSCS*													+					
*STCI-S*							+											
Other																		
Breath rate			+															
Cortisol, IL-6, CRP													+					

## 4. Discussion

Work-related stress is a growing concern for healthcare professionals. In fact, more than 20% of European workers report suffering from stress related to the workplace. Moreover, work-related stress has been associated with a number of negative health outcomes, including cardiovascular diseases, musculoskeletal disorders (particularly back problems), and absenteeism. Work-related stress develops because a person is unable to cope with work demands [63]. Factors causing work stress include poor communication or cooperation within the organization and lack of control over work pace or work processes. Protective factors for reducing work-related stress have also recently been identified, including resilience and sense of coherence (SOC). In particular, people reporting high levels of SOC have an opinion on reality and their environment that is more comprehensive, manageable, and meaningful [64].

Work-related stress can have a negative impact on the workers themselves, patients, communities, and the general population at large. Furthermore, burnout and work-related stress in healthcare professionals is associated with work-to-family conflict and unrealistic expectations of patients, which are factors that have been worsened during the pandemic [65]. 

The selected articles highlight that high levels of work-related stress and burnout can cause a significant reduction in life and job satisfaction, worsening quality of life, and can lead to the onset of psychiatric disorders such as depression. In fact, many authors use assessment tools for quality of life, job satisfaction, and depressive symptoms to evaluate the effectiveness of interventions aimed at reducing work-related stress.

Therefore, the implementation and dissemination of preventive interventions for work-related stress represents an urgent priority from a public health perspective [66,67,68]. Based on the present systematic review, different interventions are currently available for addressing work-related stress in healthcare professionals. Studies included in this review are very heterogeneous in terms of assessment tools, target populations, and types of interventions, thus limiting the generalizability of the findings. 

Among interventions focused on the individual level using CBT or other psychotherapeutic approaches, our findings confirm the effectiveness of mindfulness techniques in reducing work-related stress [46,54,59,60], which was already shown in healthy adults and children [69,70]. Specific features of mindfulness can be beneficial for preventing stress in the workplace: it can be easily practiced after a short training and it does not require particular tools nor settings, and it is usually well accepted by participants. The apparently conflicting data by Watanabe et al., could be due to the peculiarities of the specific interventions (techniques, duration, environment) and the different study design [45]. 

The main limitations of the included interventions are related to the extreme heterogeneity and to the short-term evaluation of their effectiveness. Therefore, it should be needed to promote further rigorous longitudinal studies, aiming to assess the preventive and protective effects of these interventions in the long-term. Encouraging results in reducing stress, anxiety, and depressive symptoms, as well as emotional exhaustion, are seen with other psychotherapeutic approaches, such as ESRT, EMDR, and ACT [48,49,58]. Also, referring to these latter approaches, there is not a specific protocol of intervention that is work-related stress oriented. Although CBT-based techniques may be useful in mitigating stress and promoting coping and resilience, further research to investigate the effects of SOC-strengthening interventions may be useful, since SOC has been shown to be a work-related stress-specific protective factor. [64]

As regards relaxation techniques, a promising intervention carried out by Dincer and Inangil utilizing Emotional Freedom Techniques (EFT) led to a significant reduction in stress, anxiety, and burnout levels [56]. EFT had already been shown to be effective in reducing depressive symptoms [71] and in improving stress-related conditions such as tension-type headaches [72]. Instead, Cunningham and Çayir, while reporting an efficacy in reducing the anxiety at group sessions of daylong resilience retreats, did not use any validated tools but a self-produced questionnaire [61]. On the other hand, Kaimal et al. measured the effectiveness of art-based relaxation techniques in reducing stress, but these data were not supported by changes in biomarkers, suggesting a potential placebo effect and suggesting that further studies are needed [53]. Similarly, two RCTs [55,62] found a small effectiveness for interventions based on relaxation techniques that seek to reduce and prevent burnout. Overall, relaxation techniques can be considered effective in reducing stress and can be easily applied in the workplace; in particular, they are simple techniques that do not require special training conducted by qualified personnel, are cost-effective, and can be repeated over time. On the other hand, the need to repeat the interventions several times with the same people in the workplace could represent a limitation in itself. 

### Limitations

The present review has some limitations which must be acknowledged. First, the search strategy has been limited to healthcare professionals in general, without a specific comparison among the different professional roles of participants. In fact, it could be that protective and risk factors for specific groups of professionals, such as early career professionals or nurses, are completely different from those relevant for senior medical doctors. Furthermore, the work environment, the type of patients, the tasks required, the level of responsibility, and the career opportunities available significantly change for each category and can play a role in determining the risk of developing workplace-related stress or burnout symptoms. Another limitation is due to the extreme heterogeneity of the assessment tools used for measuring the levels of workplace-related stress. This could be due to the fact that “workplace-related stress” is a complex phenomenon, including both structural and organizational factors as well as personal dimensions, such as coping strategies, temperament traits, and cognitive styles. Therefore, a complex phenomenon cannot be measured by a unique assessment tool. Finally, most of the interventions were conducted with volunteers and control groups not clearly defined. This may have led to a selection bias and to a “placebo” effect. However, the difficulty in selecting participants among healthcare professionals may explain the lack of RCTs on the subject, despite the growing interest of the scientific community.

## 5. Conclusions

The findings of the present systematic review clearly highlight the complexity of the management and prevention of work-related stress, which requires a multicomponent and multilevel approach. Despite the growing interest in the topic, it is not possible to draw definite conclusions on the “best practice” to adopt in order to prevent work-related stress among healthcare workers. It can be useful to run randomized controlled trials examining the most promising intervention techniques (such as mindfulness), which need to be well-structured and reliable. Interventions should be carried out on restricted categories of healthcare professionals, taking into account age, tasks, and type of treated patients. It is also necessary to define which assessment tools shall be used in order to compare more objectively the results and to investigate all the dimensions of burnout [73,74].

## Data Availability

Upon request to the corresponding author.

## Supplementary Materials

The following supporting information can be downloaded at: https://www.mdpi.com/article/10.3390/medicina59101866/s1, Table S1. Risk of bias assessment in non-randomized studies of intervention (NRSI). Table S2. Risk of bias assessment in randomized clinical trials (RCTs).

## References

[1] Leka S., Cox T., Griffiths A. (2003). Work Organization and Stress: Systematic Problem Approaches for Employers, Managers and Trade Union Representatives.

[2] Karam E., Kovess Masfety V. (2022). We Share More Attributes than We Think: The Crucial Input of Epidemiology. World Psychiatry.

[3] Bryant C., Fairbrother G., Fenton P. (2000). The Relative Influence of Personal and Workplace Descriptors on Stress. Br. J. Nurs..

[4] Maslach C., Schaufeli W.B., Leiter M.P. (2001). Job Burnout. Annu. Rev. Psychol..

[5] Bui T., Zackula R., Dugan K., Ablah E. (2021). Workplace Stress and Productivity: A Cross-Sectional Study. Kans. J. Med..

[6] World Health Organization International Statistical Classification of Diseases and Related Health Problems (11th ed.). https://icd.who.int/en.

[7] Parker G., Tavella G. (2022). Burnout: A Case for Its Formal Inclusion in Classification Systems. World Psychiatry.

[8] Restrepo J., Lemos M. (2021). Addressing Psychosocial Work-Related Stress Interventions: A Systematic Review. WOR.

[9] Thornicroft G. (2022). Psychiatric Diagnosis and Treatment in the 21st Century: Paradigm Shifts or Power Shifts?. World Psychiatry.

[10] Weigl T., Tölle A.-S., Seppelfrick T. (2021). Differential Aspects of Chronic Work-Related Stress Predict Depression in Registered and Geriatric Nurses: A Cross-Sectional Study. Pflege.

[11] Jovanović N., Podlesek A., Volpe U., Barrett E., Ferrari S., Rojnic Kuzman M., Wuyts P., Papp S., Nawka A., Vaida A. (2016). Burnout Syndrome among Psychiatric Trainees in 22 Countries: Risk Increased by Long Working Hours, Lack of Supervision, and Psychiatry Not Being First Career Choice. Eur. Psychiatry.

[12] Ferrari S., Cuoghi G., Mattei G., Carra E., Volpe U., Jovanovic N., Beezhold J., Rigatelli M., Galeazzi G.M., Pingani L. (2015). Young and Burnt? Italian Contribution to the International BurnOut Syndrome Study (BOSS) among Residents in Psychiatry. Med. Lav..

[13] Mulugeta H., Tamene A., Ashenafi T., Thygerson S.M., Baxter N.D. (2021). Workplace Stress and Associated Factors among Vehicle Repair Workers in Hawassa City, Southern Ethiopia. PLoS ONE.

[14] González-Siles P., Martí-Vilar M., González-Sala F., Merino-Soto C., Toledano-Toledano F. (2022). Sense of Coherence and Work Stress or Well-Being in Care Professionals: A Systematic Review. Healthcare.

[15] Alimoradi Z., Ohayon M.M., Griffiths M.D., Lin C.-Y., Pakpour A.H. (2022). Fear of COVID-19 and Its Association with Mental Health-Related Factors: Systematic Review and Meta-Analysis. BJPsych Open.

[16] Aymerich C., Pedruzo B., Pérez J.L., Laborda M., Herrero J., Blanco J., Mancebo G., Andrés L., Estévez O., Fernandez M. (2022). COVID-19 Pandemic Effects on Health Worker’s Mental Health: Systematic Review and Meta-Analysis. Eur. Psychiatry.

[17] Fiorillo A., Sampogna G., Giallonardo V., Del Vecchio V., Luciano M., Albert U., Carmassi C., Carrà G., Cirulli F., Dell’Osso B. (2020). Effects of the Lockdown on the Mental Health of the General Population during the COVID-19 Pandemic in Italy: Results from the COMET Collaborative Network. Eur. Psychiatry.

[18] Geddes J.R. (2021). Learning from the global response to COVID-19 to accelerate innovation in mental health trials. World Psychiatry.

[19] Schomerus G., Baumann E., Sander C., Speerforck S., Angermeyer M.C. (2021). Some good news for psychiatry: Resource allocation preferences of the public during the COVID-19 pandemic. World Psychiatry.

[20] Gold J.A. (2020). COVID-19: Adverse Mental Health Outcomes for Healthcare Workers. BMJ.

[21] Raudenská J., Steinerová V., Javůrková A., Urits I., Kaye A.D., Viswanath O., Varrassi G. (2020). Occupational Burnout Syndrome and Post-Traumatic Stress among Healthcare Professionals during the Novel Coronavirus Disease 2019 (COVID-19) Pandemic. Best Pract. Res. Clin. Anaesthesiol..

[22] Coleman B., Casiraghi E., Blau H., Chan L., Haendel M.A., Laraway B., Callahan T.J., Deer R.R., Wilkins K.J., Reese J. (2022). Risk of New-Onset Psychiatric Sequelae of COVID-19 in the Early and Late Post-Acute Phase. World Psychiatry.

[23] Volkow N.D., Maua S., Campello G., Poznyak V., Krupchanka D., Kashino W., Busse A. (2022). Prevention, Treatment and Care of Substance Use Disorders in Times of COVID-19. World Psychiatry.

[24] Greenberg N., Rafferty L. (2021). Post-Traumatic Stress Disorder in the Aftermath of COVID-19 Pandemic. World Psychiatry.

[25] Sampogna G., Di Vincenzo M., Giallonardo V., Perris F., Volpicelli A., Del Vecchio V., Luciano M., Fiorillo A. (2022). The Psychiatric Consequences of Long-COVID: A Scoping Review. J. Pers. Med..

[26] Mei Q., Wang F., Bryant A., Wei L., Yuan X., Li J. (2021). Mental Health Problems among COVID-19 Survivors in Wuhan, China. World Psychiatry.

[27] Carmassi C., Tosato S., Bertelloni C.A., Pedrinelli V., Cappelli A., Abbate-Daga G., Albert U., Castellini G., Luciano M., Menchetti M. (2022). PTSD Trajectories across Different Mental Disorders in the Second Year of the COVID-19 Pandemic in Italy: A Naturalistic, Longitudinal, Multicenter Study. Int. Rev. Psychiatry.

[28] Sani G., Janiri D., Moccia L., Albert U., Carrà G., Carmassi C., Cirulli F., Dell’Osso B., Menculini G., Nanni M.G. (2022). Psychopathological Burden and Coping Strategies among Frontline and Second-Line Italian Healthcare Workers Facing the COVID-19 Emergency: Findings from the COMET Collaborative Network. J. Affect. Disord..

[29] Motillon-Toudic C., Walter M., Séguin M., Carrier J.-D., Berrouiguet S., Lemey C. (2022). Social Isolation and Suicide Risk: Literature Review and Perspectives. Eur. Psychiatry.

[30] Carmassi C., Foghi C., Dell’Oste V., Cordone A., Bertelloni C.A., Bui E., Dell’Osso L. (2020). PTSD Symptoms in Healthcare Workers Facing the Three Coronavirus Outbreaks: What Can We Expect after the COVID-19 Pandemic. Psychiatry Res..

[31] Luo M., Guo L., Yu M., Jiang W., Wang H. (2020). The Psychological and Mental Impact of Coronavirus Disease 2019 (COVID-19) on Medical Staff and General Public—A Systematic Review and Meta-Analysis. Psychiatry Res..

[32] Lasalvia A., Bonetto C., Porru S., Carta A., Tardivo S., Bovo C., Ruggeri M., Amaddeo F. (2021). Psychological Impact of COVID-19 Pandemic on Healthcare Workers in a Highly Burdened Area of North-East Italy. Epidemiol. Psychiatr. Sci..

[33] Ahrens K.F., Neumann R.J., Kollmann B., Plichta M.M., Lieb K., Tüscher O., Reif A. (2021). Differential Impact of COVID-Related Lockdown on Mental Health in Germany. World Psychiatry.

[34] Steger M.F. (2022). Meaning in Life Is a Fundamental Protective Factor in the Context of Psychopathology. World Psychiatry.

[35] Turner B.J. (2022). Detecting and Managing Non-Suicidal Self-Damaging Behaviors. World Psychiatry.

[36] Klonsky E.D., Dixon-Luinenburg T., May A.M. (2021). The Critical Distinction between Suicidal Ideation and Suicide Attempts. World Psychiatry.

[37] Holt-Lunstad J. (2021). A Pandemic of Social Isolation?. World Psychiatry.

[38] Solomonov N., Kanellopoulos D., Grosenick L., Wilkins V., Goldman R., Ritholtz S., Falk A., Gunning F.M. (2022). CopeNYP: A Brief Remote Psychological Intervention Reduces Health Care Workers’ Depression and Anxiety Symptoms during COVID-19 Pandemic. World Psychiatry.

[39] Markowitz J.C. (2022). In Support of Supportive Psychotherapy. World Psychiatry.

[40] Corrigan P.W. (2022). Coming out Proud to Erase the Stigma of Mental Illness. World Psychiatry.

[41] Ruotsalainen J.H., Verbeek J.H., Mariné A., Serra C. (2015). Preventing Occupational Stress in Healthcare Workers. Cochrane Database Syst. Rev..

[42] Liberati A., Altman D.G., Tetzlaff J., Mulrow C., Gøtzsche P.C., Ioannidis J.P.A., Clarke M., Devereaux P.J., Kleijnen J., Moher D. (2009). The PRISMA Statement for Reporting Systematic Reviews and Meta-Analyses of Studies That Evaluate Healthcare Interventions: Explanation and Elaboration. BMJ.

[43] Cochrane Handbook for Systematic Reviews of Interventions. https://training.cochrane.org/handbook/current.

[44] Sterne J., Hernán M., McAleenan A., Reeves B., Higgins J. Chapter 25: Assessing Risk of Bias in a Non-Randomized Study. https://training.cochrane.org/handbook/current/chapter-25.

[45] Watanabe N., Horikoshi M., Shinmei I., Oe Y., Narisawa T., Kumachi M., Matsuoka Y., Hamazaki K., Furukawa T.A. (2019). Brief Mindfulness-Based Stress Management Program for a Better Mental State in Working Populations—Happy Nurse Project: A Randomized Controlled Trial✰✰. J. Affect. Disord..

[46] dos Santos T.M., Kozasa E.H., Carmagnani I.S., Tanaka L.H., Lacerda S.S., Nogueira-Martins L.A. (2016). Positive Effects of a Stress Reduction Program Based on Mindfulness Meditation in Brazilian Nursing Professionals: Qualitative and Quantitative Evaluation. Explore.

[47] Lebares C.C., Greenberg A.L., Ascher N.L., Delucchi K.L., Reilly L.M., van der Schaaf M., Baathe F., O’Sullivan P., Isaksson Rø K. (2021). Exploration of Individual and System-Level Well-Being Initiatives at an Academic Surgical Residency Program: A Mixed-Methods Study. JAMA Netw. Open.

[48] Luton O.W., James O.P., Mellor K., Eley C., Hopkins L., Robinson D.B.T., Lebares C.C., Powell A.G.M.T., Lewis W.G., Egan R.J. (2021). Enhanced Stress-Resilience Training for Surgical Trainees. BJS Open.

[49] Montaner X., Tárrega S., Pulgarin M., Moix J. (2022). Effectiveness of Acceptance and Commitment Therapy (ACT) in Professional Dementia Caregivers Burnout. Clin. Gerontol..

[50] West C.P., Dyrbye L.N., Satele D.V., Shanafelt T.D. (2021). Colleagues Meeting to Promote and Sustain Satisfaction (COMPASS) Groups for Physician Well-Being: A Randomized Clinical Trial. Mayo Clin. Proc..

[51] Axisa C., Nash L., Kelly P., Willcock S. (2019). Burnout and Distress in Australian Physician Trainees: Evaluation of a Wellbeing Workshop. Australas. Psychiatry.

[52] Sullivan C.E., King A.-R., Holdiness J., Durrell J., Roberts K.K., Spencer C., Roberts J., Ogg S.W., Moreland M.W., Browne E.K. (2019). Reducing Compassion Fatigue in Inpatient Pediatric Oncology Nurses. Oncol. Nurs. Forum.

[53] Kaimal G., Carroll-Haskins K., Mensinger J.L., Dieterich-Hartwell R.M., Manders E., Levin W.P. (2019). Outcomes of Art Therapy and Coloring for Professional and Informal Caregivers of Patients in a Radiation Oncology Unit: A Mixed Methods Pilot Study. Eur. J. Oncol. Nurs..

[54] Rinaldi A., Tecchio R., Perugino S., De Luca A. (2019). The Educational Intervention “Focusing” as a Strategy to Stress Reduction among Health Care Workers: A Pilot Study in an Italian Teaching Hospital. Ann. Ig..

[55] Calder Calisi C. (2017). The Effects of the Relaxation Response on Nurses’ Level of Anxiety, Depression, Well-Being, Work-Related Stress, and Confidence to Teach Patients. J. Holist. Nurs..

[56] Dincer B., Inangil D. (2021). The Effect of Emotional Freedom Techniques on Nurses’ Stress, Anxiety, and Burnout Levels during the COVID-19 Pandemic: A Randomized Controlled Trial. Explore.

[57] León-Pérez J.M., Cantero-Sánchez F.J., Fernández-Canseco Á., León-Rubio J.M. (2021). Effectiveness of a Humor-Based Training for Reducing Employees’ Distress. Int. J. Environ. Res. Public Health.

[58] Tarquinio C., Brennstuhl M.-J., Rydberg J.A., Bassan F., Peter L., Tarquinio C.L., Auxéméry Y., Rotonda C., Tarquinio P. (2021). EMDR in Telemental Health Counseling for Healthcare Workers Caring for COVID-19 Patients: A Pilot Study. Issues Ment. Health Nurs..

[59] Luzarraga J., Wichman C., Shirk R., Jarosz C., Weaver M.S. (2019). Using a Mindfulness-Based Intervention to Support the Resiliency of In-Patient Pediatric Respiratory Therapists. Respir. Care.

[60] Haghighinejad H., Ghazipoor H., Jafari P., Taghipour K., Rezaie M., Liaghat L., Ramzi M. (2022). Investigating the Impact of Modified Mindfulness-Based Stress Reduction (MBSR) Program on Occupational Burnout and Other Mental Health Status among Nonmedical Staff in a Hospital: A Randomized Controlled Trial. Int. Arch. Occup. Environ. Health.

[61] Cunningham T., Çayir E. (2021). Nurse Leaders Employ Contemplative Practices to Promote Healthcare Professional Well-Being and Decrease Anxiety. J. Nurs. Adm..

[62] Kavanaugh J., Hardison M.E., Rogers H.H., White C., Gross J. (2022). Assessing the Impact of a Shinrin-Yoku (Forest Bathing) Intervention on Physician/Healthcare Professional Burnout: A Randomized, Controlled Trial. Int. J. Environ. Res. Public Health.

[63] Ehring T. (2021). Thinking Too Much: Rumination and Psychopathology. World Psychiatry.

[64] del-Pino-Casado R., Espinosa-Medina A., López-Martínez C., Orgeta V. (2019). Sense of Coherence, Burden and Mental Health in Caregiving: A Systematic Review and Meta-Analysis. J. Affect. Disord..

[65] Izdebski Z., Kozakiewicz A., Białorudzki M., Dec-Pietrowska J., Mazur J. (2023). Occupational Burnout in Healthcare Workers, Stress and Other Symptoms of Work Overload during the COVID-19 Pandemic in Poland. Int. J. Environ. Res. Public Health.

[66] Kestel D. (2022). Transforming Mental Health for All: A Critical Role for Specialists. World Psychiatry.

[67] Campion J., Javed A. (2022). WPA Working Group on Public Mental Health: Objectives and Recommended Actions. World Psychiatry.

[68] Patton G.C., Raniti M., Reavley N. (2021). Rediscovering the Mental Health of Populations. World Psychiatry.

[69] De Vibe M., Bjørndal A., Tipton E., Hammerstrøm K., Kowalski K. (2012). Mindfulness Based Stress Reduction (MBSR) for Improving Health, Quality of Life, and Social Functioning in Adults. Campbell Syst. Rev..

[70] Paulus M.P. (2016). Neural Basis of Mindfulness Interventions That Moderate the Impact of Stress on the Brain. Neuropsychopharmacology.

[71] Nelms J.A., Castel L. (2016). A Systematic Review and Meta-Analysis of Randomized and Nonrandomized Trials of Clinical Emotional Freedom Techniques (EFT) for the Treatment of Depression. EXPLORE.

[72] Bougea A.M., Spandideas N., Alexopoulos E.C., Thomaides T., Chrousos G.P., Darviri C. (2013). Effect of the Emotional Freedom Technique on Perceived Stress, Quality of Life, and Cortisol Salivary Levels in Tension-Type Headache Sufferers: A Randomized Controlled Trial. Explore.

[73] McDaid D. (2021). Viewpoint: Investing in Strategies to Support Mental Health Recovery from the COVID-19 Pandemic. Eur. Psychiatry.

[74] Ng R.M.K. (2022). WPA Educational Initiatives: Reaching Different Stakeholders in the Mental Health Field. World Psychiatry.

