# Supplementary material for: Organizational and Individual Interventions for Managing Work-Related Stress in Healthcare Professionals: A Systematic Review
Source: Medicina (Kaunas). 2023 Oct 20;59(10):1866. doi: 10.3390/medicina59101866 (PMC10608642; doi:10.3390/medicina59101866)
Supplement: Supplementary file 1 [file medicina-59-01866-s001.zip › medicina-2619029-supplementary.pdf]

Supplementary Table S1. *Risk of bias assessment in non-randomized studies of intervention (NRSI).*

First Author (year of publication)	*Pre-intervention domains*		*At-intervention domain*	*Post-intervention domains*				Overall risk of bias
	Confounding bias	Selection bias	Information bias	Confounding bias	Selection bias	Information bias	Reporting bias	
Dos Santos (2015)	Low	Low	Low	Moderate	Low	Moderate	Low	Moderate
Kaimal (2019)	High	Moderate	Moderate	Moderate	Low	Moderate	Moderate	High
Luzarraga (2019)	High	Moderate	Moderate	Moderate	High	Moderate	Moderate	High
Rinaldi (2019)	High	Low	Moderate	Moderate	Moderate	Moderate	Moderate	High
Sullivan (2019)	Low	Low	Moderate	Moderate	Moderate	Moderate	Moderate	Moderate
Cunningham (2021)	Moderate	Low	High	Moderate	Low	Moderate	Moderate	High
Lebares (2021)	High	Moderate	Low	Moderate	Moderate	Moderate	Low	High
León-Pérez (2021)	Moderate	Moderate	Moderate	Moderate	Low	Moderate	Low	High
Luton (2021)	Low	Moderate	Low	Moderate	High	Moderate	Moderate	High
Tarquinio (2021)	Low	High	Low	Moderate	Low	Moderate	Low	High

Supplementary Table S2. *Risk of bias assessment in randomized clinical trials (RCTs).*

First Author (year of publication)	Randomization process	Deviation from intended intervention	Missing outcome data	Measurement of the outcome	Selection of the reported results	Overall risk of bias
Calder Calisi (2017)	Low	Moderate	Low	Moderate	Low	Moderate
Axisa (2019)	Low	Moderate	Low	Moderate	Low	Moderate
Watanabe (2019)	Low	Low	Low	Low	Low	Low
Dincer (2020)	Low	Moderate	Low	Moderate	Low	Moderate
Montaner (2021)	Low	Moderate	Low	Moderate	Low	Moderate
West (2021)	Low	Moderate	Low	Moderate	Low	Moderate
Haghighinejad (2022)	Low	Moderate	Low	Moderate	Low	Moderate
Kavanaugh (2022)	Low	Moderate	Low	Moderate	Low	Moderate
